# Inter-examinerreliability study of physical examination procedures to assess the cervical spine

**DOI:** 10.1186/s12998-021-00377-2

**Published:** 2021-06-14

**Authors:** Karthik V. Hariharan, Michael G. Timko, Christopher G. Bise, Meenakshi Sundaram, Michael J. Schneider

**Affiliations:** 1 Department of Physical Therapy, University of Pittsburgh, Pittsburgh, PA, USA; 2 Division of Physical Therapy, West Virginia University, Morgantown, WV USA; 3 Clinical and Translational Science Institute, University of Pittsburgh, Pittsburgh, PA USA

**Keywords:** ***Cervical spine***, ***physical examination***, ***palpation***, ***neck pain***, ***reliability***

## Abstract

**Objective:**

The objective of this study was to establish the level of inter-examiner reliability for six common cervical manual and physical examination procedures used to assess the cervical spine.

**Materials:**

Reliability study that used a convenience sample of 51 patients between the ages of 16–70 years presenting with a chief complaint of neck pain. Two physical therapists independently performed the same series of cervical physical examination procedures on each of the participant. The clinicians were blinded to each other’s findings and the clinical status of the patient. Kappa coefficients (κ) were calculated for levels of agreement between the clinicians for each procedure.

**Results:**

When assessing for asymmetrical motion, excellent levels of reliability (κ range: 0.88–0.96) were observed for the Bilateral Modified Lateral Shear (asymmetry criterion), Bilateral C2 Spinous Kick (asymmetry criterion) and Flexion-Rotation Tests. When pain provocation was used as the indicator of a positive test during palpation of the cervical facet joints, moderate to substantial levels of reliability (κ range: 0.53–0.76) were observed. When patients were instructed not to provide feedback to the clinicians about pain provocation during facet joint palpation and clinicians relied solely on their qualitative assessment of segmental mobility, the level of reliability was lower (κ range: 0.45–0.53). Due to 100 % prevalence of negative findings, Kappa values could not be calculated for the Sharp-Purser test or the Unilateral C2 Spinous Kick Test.

**Conclusions:**

Most physical examination procedures examined in this study demonstrated moderate to excellent levels of inter-examiner reliability. Palpation for segmental mobility without pain provocation demonstrated a lower level of reliability compared to palpation for pain provocation. Correlation with clinical findings is necessary to establish validity and the applicability of these procedures in clinical practice.

## Introduction

Neck pain is a common musculoskeletal disorder with an annual prevalence exceeding 30 % and is considered the fourth leading cause of disability worldwide [1, 2]. Like low back pain, most cases of neck pain are considered to be “non-specific”, because a specific pathoanatomical source of the pain cannot typically be determined. Non-specific neck pain is also one of the most common clinical conditions for which patients seek chiropractic or physical therapy care [3].

The majority of patients who seek chiropractic or physical therapy treatment for neck pain do not exhibit red flags of serious pathology or neurologic deficit in the standard clinical assessment, that involves taking a comprehensive case history to rule out serious illness or injury and physical examination that includes physical and neurological tests [4]. Instead, the cause of the neck pain is thought to be “mechanical”; associated with some type of muscle and/or joint dysfunction that can be treated with manual therapy, exercise and posture education [5]. There are many “mechanical” assessments used by chiropractors and physical therapists to determine localized areas of musculoskeletal pain and dysfunction in the cervical spine [6]. These procedures involve two components: (1) pain provocation and (2) qualitative assessments by the clinician about the perceived amount of joint motion and muscle tone [7].

Mechanical examination procedures of the cervical facet joints involve active and passive range of motion (ROM) testing where the examiner is looking for the quality of the observed motion for symmetry and pain provocation and applies various types of manual overpressure to the cervical vertebrae to assess the quality and symmetry of intersegmental joint motion [6]. Manual palpation is also utilized to assess for the quality of localized muscle tone in order to screen for the presence of taut bands or myofascial trigger points. Several systematic reviews of reliability studies of movement and palpation tests in patients with neck pain have been published, which report mixed conclusions with inconsistent reliability estimates found from different individual reliability studies [8–10]. A recent systematic review found little evidence to support the reliability and validity of most clinical tests used by physical therapists and chiropractors to assess head posture, pain location and cervical mobility in patients with neck pain [11]. Another recent systematic review found inconsistent levels of reliability for movement and pain testing procedures ranging from poor to almost perfect, with the overall conclusion that passive intervertebral tests had poor reliability [12].

The main reason for these inconsistencies found in the literature regarding the reliability of movement and palpation procedures is that many of the individual reliability studies have been of low quality with a high risk of bias [10, 11]. Yet many of these procedures continue to be commonly employed by physical therapists and chiropractors as clinical indicators for manual therapy treatment and as a means of assessing response to treatment.

The systematic review mentioned above that found inconsistencies in levels of reliability also found that palpation tests for pain assessment in the segments of the cervical spine tended to show overall more acceptable reliability than assessments of passive movement (segmental mobility) [10]. This highlights a gap in the literature; that few studies have been conducted in which both pain provocation and segmental mobility tests have been performed on the same set of patients, with head-to-head comparisons of their respective levels of reliability. To address this gap, we designed a reliability study to primarily to compare muscle and joint palpation procedures that are based upon objective pain responses with those based upon subjective judgements of segmental mobility and muscle tone. We also included some other manual palpation procedures that are commonly used to assess the joints of the upper cervical spine (cranio-cervical junction).

## Methods

This was an inter-examiner reliability study that used a convenience sample of 51 participants (33 females, 18 males) over the age of 16 years with a history of neck injury with lingering pain and symptoms. Power calculation indicated the need for a minimum sample size of 40 participants, which would provide 80 % power (alpha = 0.05) to detect a Kappa as low as 0.40, which is a reliability value in the “fair to moderate” range. In order to account for potential drop-outs or no-shows, we decided that it would be prudent to use a slightly larger convenience sample, which led to an increase in our sample size to 51 participants.

Our rationale for recruiting patients with neck pain due to injury was pragmatic, we simply wanted to ensure that our participants were actively experiencing neck pain. We recognize that injured patients with subacute and chronic neck pain differ from non-injured patients with acute neck pain on certain baseline characteristics. However, our study was only concerned with determining the reliability of testing procedures and not the clinical characteristics of the participants. Participants were recruited from multiple physical medicine and physical therapy clinics in Pittsburgh, Pennsylvania, USA. All participants were screened for eligibility and informed consent was obtained prior to inclusion in the study. The study was approved by the University of Pittsburgh Institutional Review Board (PRO13050068). No internal or external funding was provided for this study; all participants, examiners and researchers were volunteers.

Participants were considered for inclusion if they met the following criteria: age greater than or equal to 16 years; history of closed head and/or neck injury; and symptoms of neck pain and/or headache that had been lingering for a minimum of 3 weeks. Participants were excluded if they were pregnant, had a history of cancer, had radiographic evidence of fracture/dislocation of the cervical spine, or had a history of cervical spine surgery.

All participants were informed that they would have their neck examined by two different physical therapists, while a member of the research team observed the examination and recorded the findings. After consenting to participation, the participants underwent the same series of six cervical spine physical examination procedures performed by two different licensed physical therapists (PTs) on the same day. Two PTs independently examined each participant and were blinded to each other’s findings, as well as the participants’ clinical status. Each research participant was bought into a private room in our physical therapy research center and briefed regarding what to expect during the study. Written informed consent was then obtained. The first PT entered the room and proceeded to examine the research participant while a research team member observed and recorded the findings. The second PT then came into the room and repeated the same six examination procedures, blinded to the first PT’s findings.

The study involved a total of seven PTs who all had previous clinical experience with the use of these examination procedures. To ensure consistency with the application of all examination procedures, all seven PTs were required to complete a 1-hour in-person review and training session of all test procedures with a research investigator. A pool of multiple PTs was needed to provide wide flexibility in our ability to schedule two PTs at the same day/time to examine research participants. The determination of which two PTs examined a research participant was based upon their availability, and their ability to meet the scheduling needs of the participant. This led to multiple pairings of different PTs from our pool of seven PTs, which we considered would increase the generalizability of our findings.

This study included six different physical examination procedures/tests commonly used by manual therapists to assess the soft tissues and joints of the cervical spine:

1. Sharp Purser Test [13, 14]
2. Flexion Rotation Test [13, 15]
3. C2 Spinous Kick Test [13].
4. Modified Lateral Shear Test [13].
5. Palpation of sub-occipital muscles for taut bands and pain provocation [7, 11, 16]
6. Palpation of cervical facet joints for segmental mobility and pain provocation [7, 11, 16]

The findings of all six physical examination procedures were recorded as dichotomous variables. Table 1 provides a description of how each test was performed and the criteria used for determining positive/negative test results. The result of the Sharp Purser and Flexion Rotation tests were recorded simply as ‘positive’ or ‘negative’. For the C2 spinous kick test and modified lateral shear test, the examiners were asked to report their findings in two ways: (1) the examiner provided a judgement as to whether the movements they palpated were ‘symmetrical’ or ‘asymmetrical’ and (2) the examiner was asked to determine if each unidirectional movement of C1 or C2 (to the left and to the right) was ‘normal’ or ‘abnormal’.

**Table 1:** Descriptions of the physical examination procedures and definitions for a positive test

Procedure	Description of procedure and definition for a positive test
Sharp Purser [13, 14]	This procedure screens for integrity of the transverse portion of the cruciform ligament and/or fracture of the Dens of C2 in the sagittal plane. Palpation of posterior movement of the head and relocation (“clunk”) of C1 on C2 is considered a positive test.
Unilateral C2 Spinous Kick Test [13, 14]	This procedure screens for the coupled rotation of C2 during cervical lateral bending. Palpation of C2 spinous rotation contralateral (away) from the direction of side bending is considered normal. A positive test is when the examiner palpates a lack of C2 spinous rotation during lateral bending to one side.
Bilateral C2 Spinous Kick Test (asymmetry criterion)	Same procedure as above but comparing the amount of C2 rotation from one side to the other. A positive test is when the examiner notes an asymmetry in the amount of contralateral C2 spinous movement (kick) during side-bending.
Flexion Rotation Test [13, 14]	With the neck in full flexion, the examiner passively rotates the head bilaterally. A positive test is when the examiner observes an asymmetry in amount of rotation side to side, or symptoms are provoked by rotation to one or both sides.
Unilateral Modified Lateral Shear Test [13, 14]	This procedure screens for integrity of the alar ligaments in the coronal plane. Palpation of lateral translation of the C1 transverse process in relationship to C2 is considered a positive test. i.e. a firm end feel during translation is considered a negative test and a unilateral loose or ‘mushy’ end feel is considered a positive test.
Bilateral Modified Lateral Shear Test (asymmetry criterion)	Same procedure as above but in this case, an assessment of asymmetry of end feel during lateral translation is considered a positive test.
Sub-occipital muscle palpation with verbal pain provocation	Patient report of tenderness or pain provocation during muscle palpation of the sub-occipital region is considered a positive test.
Sub-occipital muscle palpation for presence of taut bands without verbal pain provocation	Taut bands or tightness assessed by the therapist during palpation of sub-occipital muscles is considered a positive test. Patients were asked not to verbalize pain or tenderness during this procedure.
Facet joint mobility testing: mid-cervical and lower-cervical with verbal pain provocation	Patient report of pain or tenderness provoked during palpation of the facet joints in the mid and lower cervical spine is considered a positive test.
Facet joint mobility testing: mid- and lower-cervical segmental mobility without verbal pain provocation	An assessment by the therapist of restricted segmental mobility during palpation is considered a positive test. Patients were asked not to verbalize pain or tenderness during this procedure.

For the unidirectional C2 spinous kick test, normal movement was defined as feeling the C2 spinous process rotate (‘kick’) to the side opposite of lateral bending. For the unilateral Modified Lateral Shear Test, normal motion was defined as a firm end-feel (no laxity) when applying lateral pressure to the C1 transverse process in the frontal plane.

Muscle palpation was performed with the patient lying supine on an examination table, with the examiner using static manual pressure to assess the sub-occipital muscles for taut or hypertonic muscle fibers. The presence of pain during testing was documented when the patient provided verbal feedback to the clinician. Facet joint palpation was performed with the patient supine and the clinician applying manual posterior to anterior pressure (‘springing’ palpation) over the facet joints, in a sagittal plane vector. Theoretically, this manual overpressure introduces segmental motion into the targeted facet joints [7]. The examiners were asked to assess the segmental mobility of the palpated facet joints and record their judgement as to whether they perceived the joint motion to be ‘normal’ or ‘restricted’. They also recorded the presence of pain provocation during testing as indicated by verbal patient response during the segmental joint palpation procedure. We defined mid-cervical facet joints as the C2-3, C3-4 and C4-5 motion segments, and lower-cervical spine facet joints as the C5-6, C6-7 and C7-T1 segments.

The muscle and facet joint palpation procedures were performed twice (two applications) by each examiner on each research participant on the same day. During the first application of palpation, the participants were told not to indicate to the examiner whether pain was being provoked during the palpation. Each examiner was asked to report their own subjective assessment of muscle tone as ‘normal’ or ‘increased’ and segmental joint mobility assessments as ‘normal’ or ‘restricted’, based solely upon what they discerned or perceived with their hands. After the research team member recorded the subjective assessments of suboccipital muscle tone and segmental mobility, a second application of the same palpation procedures were repeated by the same examiner. However, this time participants were asked to indicate whenever pain was provoked during the muscle and joint palpation procedures, with a research team member recording this additional set of muscle and joint palpation findings as ‘pain’ or ‘no pain’. A research team member was present for all examinations and observed to make sure the patient followed these instructions and that the examination procedures were performed according to the protocol.

### Data Analysis

Data were analyzed using SPSS v23, which included calculations of standard Cohen’s unadjusted Kappa values with their respective 95 % confidence intervals (CIs), raw percentages of agreement, prevalence index, bias index, and raw numbers of positive/negative findings for each examiner. The prevalence index is the absolute difference between concordant agreements on positive/negative findings divided by the total number of subjects [17]. The bias index is calculated in a similar manner but is based upon the discordant agreements on positive and negative findings.

Both the prevalence and bias index values may affect calculation of the Kappa statistic, especially when the prevalence or bias index values are greater than 0.5. Since several tests were associated with high prevalence index values (> 0.5), we also calculated Prevalence And Bias Adjusted Kappa (PABAK) values with their respective 95 % CIs [7]. These PABAK values help to reduce the potential confounding of the high prevalence of positive/negative results of certain testing procedures.

### Interpretation of Kappa statistic

Cohen’s Kappa (unadjusted) and the PABAK (adjusted) statistic both reflect the level of agreement between examiners that is above chance agreement. Kappa values range from 0.0 to 1.0, with 0.0 indicating pure chance (50 % raw agreement) and 1.0 indicating perfect agreement (100 % raw agreement). The PABAK statistic is considered a more robust measure of reliability, because it adjusts for the prevalence of positive/negative findings. Kappa values of 0.21 to 0.40 are considered “fair”, values between 0.41 and 0.60 are considered “moderate”, values of 0.61 to 0.80 are considered “substantial”, and values greater than 0.80 are considered “excellent” [17, 18]. Some tests with a lower level of Kappa values still may still be valuable for clinical decision making. Therefore, some have proposed a minimal acceptable cutoff score of Kappa greater than 0.40 for tests used in the clinical setting [19]. A simple way to interpret values is to think of the decimal value as the percentage of agreement above chance. For example, a Kappa value of 0.5 indicates a level of agreement that is 50 % better than chance alone.

## Results

Table 2 contains the demographic information regarding the participants (*n* = 51) and the physical therapists who served as examiners (n = 7) in this study. Table 3 provides a summary of the results, including calculations of Cohen’s unadjusted Kappa and Prevalence And Bias Adjusted Kappa (PABAK) values with their respective 95 % confidence intervals (CIs). We also included raw percentages of agreement, prevalence and bias indices, as well as the proportion of negative/positive tests found by each of the two physical therapists. As stated earlier, the PABAK statistic is considered a more robust measure of reliability, because it adjusts for the prevalence of positive/negative findings. Therefore, we only refer to the PABAK values in the narrative descriptions of the interpretation of our study results [17–19].

**Table 2:** Demographic information about participants (*n* = 51) and examiners (*n* = 7)

Characteristic	Mean (SD)	Range
**Participants (*n* = 51)**		
Age (years)	33 (± 18.8)	16–71
Sex (female/male)	33/18	-
Duration of symptoms (days)	67 (± 50.5)	23–304
**Examiners (*n* = 7)**		
Clinical experience (years)	4.9 (± 4.7)	0.5–15
Sex (female/male)	3/4	-
Number of examinations performed by each examiner (either as examiner #1 or #2)	28.9 (± 9.0)	19–38

**Table 3:** Results for cervical spine physical examination procedures (*N* = 51)

Procedure	Cohen’s Kappa (unadjusted)	95 % Confidence Interval (Cohen’s K)	Raw Agreement(%)	Bias Index	Prevalence Index	Prevalence and Bias Adjusted Kappa (PABAK)	95 % Confidence Interval ^(PABAK)^	Examiner 1 (Negative/ Positive findings)	Examiner 2 (Negative/ Positive findings)
Sharp Purser	N/A^a^	N/A^a^	100	0	1	1	N/A^a^	51/0	51/0
Unilateral C2 Kick Test to left with right Side Bending	N/A^a^	N/A^a^	100	0	1	1	N/A^a^	51/0	51/0
Unilateral C2 Kick Test to right with left Side Bending	N/A^a^	N/A^a^	100	0	1	1	N/A^a^	51/0	51/0
Bilateral C2 Kick Test - Side to Side comparison (asymmetry criterion)	0.93	0.79-1.0	98	-0.02	0.67	0.96	0.92-1.0	42/9	43/8
Flexion Rotation Test	0.92	0.80-1.0	96	0.04	0.25	0.92	0.87–0.98	33/18	31/20
Unilateral Modified Lateral Shear Test (right to left)	0.31	-0.02-0.64	82	0.09	0.71	0.65	0.54–0.75	46/5	41/10
Unilateral Modified Lateral Shear Test (left to right)	0.41	0.07–0.74	84	0.04	0.69	0.69	0.59–0.79	44/7	42/9
Bilateral Modified Lateral Shear Test - Side to Side comparison (asymmetry criterion)	0.77	0.52-1.0	94	0.06	0.71	0.88	0.82–0.95	45/6	42/9
Sub-occipital muscle palpation with verbalization of pain provocation	0.64	0.43–0.85	82	-0.06	-0.12	0.65	0.54–0.75	21/30	24/27
Sub-occipital muscle palpation for ‘tightness’ without verbalization of pain provocation	0.58	0.35–0.81	80	-0.04	0.25	0.61	0.50–0.72	31/20	33/18
Segmental mobility testing with verbalization of pain provocation (^b^mid cervical spine)	0.46	0.20–0.72	76	0.08	0.37	0.53	0.41–0.65	37/14	33/18
Segmental mobility testing without verbalization of pain provocation (^b^mid cervical spine)	0.45	0.21–0.70	73	0.08	-0.02	0.45	0.33–0.57	27/24	23/28
Segmental mobility testing with verbalization of pain provocation (^c^lower cervical spine)	0.75	0.56–0.94	88	-0.04	0.25	0.76	0.68–0.85	31/20	33/18
Segmental mobility testing without Verbalization of pain provocation (^c^lower cervical spine)	0.35	0.06–0.64	76	0.08	0.53	0.53	0.41–0.65	41/10	37/14

^a^Kappa values did not compute due to 100 % prevalence of negative findings

^b^Mid cervical facet joints = C2-3, C3-4 and C4-5

^c^Lower cervical facet joints = C5-6, C6-7 and C7-T1

The Kappa values for the Sharp Purser Test and unilateral C2 Spinous Kick Tests could not be calculated due to 100 % raw agreement between the examiners. Whenever a particular observation has 100 % prevalence of either a positive or negative finding, it confounds calculation of the Kappa statistic because of a violation of the assumption of chance agreement. Our examiners did not find any patients who exhibited a positive Sharp Purser test, and consistently found that the C2 spinous process ‘kicked’ to the contralateral side during lateral bending (negative unidirectional test). Therefore, the Kappa statistic could not be calculated for these tests because there was universal agreement that these tests were always negative (0 % chance for a positive test).

Despite our inability to calculate reliability of the unidirectional C2 spinous kick test due to 100 % prevalence of a negative test, we found excellent reliability (PABAK = 0.96) when we asked the examiners to use the asymmetry criterion for determining a positive test. This involved making a comparison between the amount of C2 spinous movement they perceived during right and left lateral bending, or the perception of delayed C2 spinous rotation (‘kick’) to one side compared to the other.

We found substantial reliability (PABAK = 0.65 right; 0.69 left) for the unidirectional tests that relied upon the examiner’s judgement of whether the lateral translational motion of C1 to one side had a normal or abnormal end-feel. However, we found excellent reliability (κ = 0.88) for the bilateral test that involved the symmetry criterion; an assessment by the examiner of a positive test when making a judgement of asymmetrical motion during the comparison of right to left lateral translation. The differences in the Cohen’s Kappa and PABAK values for the unilateral modified lateral shear tests highlight the reason why the PABAK is considered to provide a more precise Kappa estimate. The Cohen’s Kappas were only considered ‘fair’ (0.31 to 0.41) with wide 95 % CIs that ranged from − 0.02 to 0.74. This meant that the real unadjusted Kappa value could lie somewhere between complete chance agreement to excellent agreement. However, the PABAK values for these tests were considered ‘substantial’ (0.65 to 0.69) with very narrow 95 % CIs that ranged from 0.54 to 0.79. This meant that the real adjusted Kappa value could lie somewhere between ‘moderate’ to ‘excellent’ agreement. (Table 3)

The results of sub-occipital muscle palpation showed substantial levels of agreement. The PABAK value was 0.61 for determination of taut bands/muscle “tightness” without patient feedback about pain provocation, and a PABAK value of 0.65 when patients reported pain provocation during muscle palpation. The reliability of facet joint palpation was generally higher when pain provocation was used as the criterion for a positive finding, as compared to using segmental mobility without feedback about pain provocation as the determination for a positive finding. The PABAK values were moderate (0.53) to substantial (0.76) for pain provocation during facet joint palpation of the mid-cervical and low-cervical regions respectively. However, PABAK values were only moderate for segmental mobility facet joint palpation without pain provocation of the mid-cervical (0.45) and low-cervical (0.53) regions. The results for segmental mobility testing without pain provocation showed a similar pattern of wide 95 % CIs for the Cohen’s Kappa values, yet narrow CIs for the PABAK values. This again reinforces the importance of using adjusted PABAK values for interpreting these results.

## Discussion

In this study, we explored the level of inter-examiner reliability for a subset of commonly utilized physical examination procedures/tests for assessing joint motion and localized muscle tone in the cervical spine. A pattern that emerged from our results from the upper cervical testing procedures; that the most reliable tests were those that utilized the finding of some type of asymmetrical movement as the criterion for a positive test. The test with the highest overall level of reliability was the flexion rotation test for asymmetrical motion, and our results are consistent with those previously reported in the literature [15]. The C2 spinous kick test and modified lateral shear test also demonstrated substantial to excellent reliability when asymmetrical motion was used as the criterion for a positive test.

There were two reasons why we chose to report both Cohen’s Kappa (unadjusted) and PABAK values.

It is important to note that any type of manual palpation is often confounded by non-verbal or verbal communication by patients. It is very difficult to separate the “subjective” pain provocation component of facet joint mobility testing from the “objective” palpatory sensations reported by clinicians [20]. However, our study design allowed us to control for the subjective component by providing explicit instructions to the research participants not to verbally communicate any of their symptoms to the examining PTs especially during the phase when the examiner was performing the palpation procedures without subjective feedback for both the examiners.

One finding of note was the level of inter-examiner reliability for segmental mobility testing. We found a lower level of reliability when using palpation to test mid cervical and lower cervical facet joints for segmental mobility, when the patients did not give verbal feedback about pain provocation. However, the reliability values increased to the moderate to substantial range for palpation of the mid and lower cervical joints when pain provocation - instead of ‘segmental restriction’ - was used as the criterion for a positive joint palpation test. We cannot rule out the potential of involuntary subjective feedback to the examiners during these procedures but believe that this would have been minimal and unavoidable. Various types of non-verbal pain provocation responses like grimacing, wincing, guarding etc. that could have been observed by the examiner and unconsciously influenced the examiner’s findings. Thereby, artificially inflating the level of inter-examiner reliability associated with these assessments of segmental mobility and muscle tightness. This possibly makes our findings more significant.

These results support the clinical observation that pain provocation is a more reliable predictor of cervical joint dysfunction than clinicians’ qualitative assessment of segmental hypomobility. Our results are also consistent with the findings from other reliability studies, which found that palpation methods based upon eliciting pain responses were more reliable than palpation methods based upon clinician assessments of segmental motion restriction, independent of the symptomatic responses reported by the patient [8, 17, 21, 22].

There is the possibility that these participants suffering from neck pain were aware of their pain site which could have led them to respond with affirmation during the pain provocation assessments making it a more consistent finding and thereby more reliable [12]. Our results cast some doubt on the assertion by some manual therapists that they can independently assess segmental joint mobility and end-feel, without verbal or non-verbal feedback from the patient about pain provocation.

Also, the results from our upper cervical tests indicate that there is higher reliability for the observation of asymmetrical motion, rather than unilateral mobility assessments. We found substantial reliability for each unilateral performance of the modified lateral shear test using soft end-feel as the positive test criterion. However, the bilateral version of the test showed excellent reliability when the positive test criterion was the qualitative assessment for asymmetrical motion from side to side.

We believe that our study results are relatively generalizable. If we had utilized only one pair of highly experienced examiners, this might have inflated their level of inter-examiner reliability. In the real-world clinical setting, the skill level of clinicians varies and so we took this into consideration by using a pool of clinicians with varying levels of clinical experience participate as examiners in our study. Different pairs of PT examiners were created based on their availability and we believe that this convenience sampling would have mitigated any confounding order effect or the lack of formal randomization.

## Clinical implications

Various types of palpatory assessments are commonly used in clinical settings to guide the application of manual therapy techniques for the management of the cervical spine conditions. Chiropractors and physical therapist use these clinical examination procedures for planning treatment as well as for reassessment purposes to check patient progress. In the literature, springing palpation or segmental mobility testing has been shown to have low inter-rater reliability despite that it has been found to be a strong predictor of clinical success. Hence, the use of segmental mobility assessment as a criterion for determining the level of cervical spine involvement has been questioned. Our results show that springing palpation is more reliable when associated with verbalization (or perhaps non-verbal communication) of pain provocation, which may help clinicians to more accurately identify areas of the cervical spine that require manual therapy. Therefore, we suggest that it may be more accurate to describe springing palpation as a pain provocation test instead of a test of segmental mobility.

## Limitations

We have established that several of these physical examination procedures have high levels of inter-examiner reliability. A limitation of our study is that we only explored the inter-examiner reliability of these procedures, but not their validity which would require a comparison of the results of these examination procedures against a ‘gold standard’ reference such as bi-plane motion radiography or traditional fluoroscopy. The other limitation of our study is that we performed the segmental mobility testing only in the sagittal plane and did not assess for symmetry of movement in lateral bending or rotation. Therefore, we missed the opportunity to identify the reliability of palpation related to examination of these other planes of motion.

Another limitation of our study was the lack of clinical measures of pain or disability with which to correlate our findings. For example, it would have been interesting to see if the patients with positive test findings were more likely to have greater levels of self-reported neck pain and disability. We also cannot rule out the possibility of a carryover effect, where the performance of the palpation procedures by the first examiner might have altered the segmental motion and pain responses found by the second examiner. Lastly, all of our patients had a history of neck injury and our results may not be generalizable to the population of non-injured patients with non-specific neck pain.

## Conclusions

This study has provided a baseline level of inter-examiner reliability for six common cervical examination procedures and tests. We could not assess the reliability of the Sharp-Purser or unilateral C2 kick tests due to lack of any positive results. The findings for several physical examination procedures showed moderate to excellent levels of inter-examiner reliability. Future research studies will be needed to establish the validity of these individual examination procedures, or clusters of these tests, in clinical populations of patients with and without various cervical diagnoses.

## Data Availability

The raw data from this study are retained in a secure database and are available to be shared with other researchers upon request and an approved data use agreement.

## References

[1] Cohen SP. Epidemiology, diagnosis, and treatment of neck pain. Mayo Clin Proc. 2015;90(2):284 – 99. 10.1016/j.mayocp.2014.09.008 25659245

[2] Murray CJ, Atkinson C, Bhalla K, Birbeck G, Burstein R, Chou D (2013). The state of US health, 1990–2010: burden of diseases, injuries, and risk factors. Jama.

[3] Nordin M, Carragee EJ, Hogg-Johnson S, Weiner SS, Hurwitz EL, Peloso PM (2009). Assessment of neck pain and its associated disorders: results of the Bone and Joint Decade 2000–2010 Task Force on Neck Pain and Its Associated Disorders. J Manipulative Physiol Ther.

[4] Pierre Côté D, PhD JD, Cassidy D, PhD, Linda Carroll P (2003). The epidemiology of neck pain:what we have learned from our population-based studies. J Can Chiropr Assoc.

[5] Blanpied PR, Gross AR, Elliott JM, Devaney LL, Clewley D, Walton DM (2017). Neck Pain: Revision 2017. J Orthop Sports Phys Ther.

[6] Triano JJ, Budgell B, Bagnulo A, Roffey B, Bergmann T, Cooperstein R (2013). Review of methods used by chiropractors to determine the site for applying manipulation. Chiropr Man Therap.

[7] Bergmann TPD (2011). Chiropractic Technique: Principles and Procedures.

[8] Hollerwöger D (2006). Methodological quality and outcomes of studies addressing manual cervical spine examinations: a review. Man Ther.

[9] Haneline MT, Cooperstein R, Young M, Birkeland K (2008). Spinal motion palpation: a comparison of studies that assessed intersegmental end feel vs excursion. J Manipulative Physiol Ther.

[10] Haneline MT, Young M (2009). A review of intraexaminer and interexaminer reliability of static spinal palpation: a literature synthesis. J Manipulative Physiol Ther.

[11] Geoff M, Schneider P, Gwendolen Jull P, PhD, Kenneth Thomas M, MHSc, Ashley Smith P, Carolyn Emery P, PhD, Peter Faris P (2013). Intrarater and Interrater Reliability of Select Clinical Tests in Patients Referred for Diagnostic Facet Joint Blocks in the Cervical Spine. Arch Phys Med Rehabil.

[12] Jonsson A, Rasmussen-Barr E (2018). Intra- and inter-rater reliability of movement and palpation tests in patients with neck pain: A systematic review. Physiother Theory Pract.

[13] Magee DJ (2014). Orthopedic Physical Assessment.

[14] Mansfield CJ, Domnisch C, Iglar L, Boucher L, Onate J, Briggs M (2020). Systematic review of the diagnostic accuracy, reliability, and safety of the sharp-purser test. J Man Manip Ther.

[15] Hall TM, Robinson KW, Fujinawa O, Akasaka K, Pyne EA (2008). Intertester reliability and diagnostic validity of the cervical flexion-rotation test. J Manipulative Physiol Ther.

[16] Lemeunier N, Jeoun EB, Suri M, Tuff T, Shearer H, Mior S (2018). Reliability and validity of clinical tests to assess posture, pain location, and cervical spine mobility in adults with neck pain and its associated disorders: Part 4. A systematic review from the cervical assessment and diagnosis research evaluation (CADRE) collaboration. Musculoskelet Sci Pract.

[17] Sim J, Wright CC (2005). The kappa statistic in reliability studies: use, interpretation, and sample size requirements. Phys Ther.

[18] Landis JR, Koch GG (1977). The measurement of observer agreement for categorical data. Biometrics.

[19] Stochkendahl MJ, Christensen HW, Hartvigsen J, Vach W, Haas M, Hestbaek L (2006). Manual examination of the spine: a systematic critical literature review of reproducibility. J Manipulative Physiol Ther.

[20] Schneider M, Erhard R, Brach J, Tellin W, Imbarlina F, Delitto A (2008). Spinal palpation for lumbar segmental mobility and pain provocation: an interexaminer reliability study. J Manipulative Physiol Ther.

[21] Cleland JA, Childs JD, Fritz JM, Whitman JM (2006). Interrater reliability of the history and physical examination in patients with mechanical neck pain. Arch Phys Med Rehabil.

[22] Piva SR, Erhard RE, Childs JD, Browder DA (2006). Inter-tester reliability of passive intervertebral and active movements of the cervical spine. Man Ther.

